# Thrombocytose au cours d'un syndrome d'activation macrophagique compliquant un lupus érythémateux systémique

**DOI:** 10.11604/pamj.2014.19.11.2071

**Published:** 2014-09-08

**Authors:** Fernando Kemta Lekpa, Souhaïbou Ndongo, Seynabou Fall, Abdoulaye Pouye, Mamadou Mourtalla Ka, Thérèse Moreira-Diop

**Affiliations:** 1 Service de Médecine Interne, Hôpital Général de Douala, Cameroun; 2 Clinique Médicale 1, CHU Aristide Le Dantec, UCAD, Dakar, Sénégal; 3 Faculté de Médecine, Université de Thiès, Thiès, Sénégal

**Keywords:** Syndrome d′activation macrophagique, Lupus érythémateux systémique, Cytopénie, Thrombocytose, Afrique Subsaharienne, macrophage activation syndrome, systemic lupus erythematosus, Cytopenia, Thrombocytosis, Sub-Saharan Africa

## Abstract

Le syndrome d'activation macrophagique (SAM) est une complication du lupus érythémateux systémique (LES), due à l'activation et la prolifération incontrôlée des macrophages dans la moelle osseuse. La bycytopénie voire la pancytopénie est constante. Nous rapportons un cas atypique de SAM diagnostiqué au même moment qu'un LES chez une patiente noire de 17 ans. Le tableau initial associait une fièvre, un syndrome inflammatoire, une anémie, un taux normal de leucocytes et plus surprenant, une thrombocytose.

## Introduction

Le syndrome d'activation macrophagique (SAM) est une complication sous estimée des maladies inflammatoires, dominée par le lupus érythémateux systémique (LES). Une bicytopénie voire une pancytopénie est classique au moment du diagnostic [1]. Nous rapportons un cas atypique de LES compliqué d'un SAM, présentant une cytopénie isolée.

## Patient et observation

Une patiente noire de 17 ans, guinéenne, avait été hospitalisée dans notre service pour une polyarthrite fébrile évoluant depuis 11 mois. L'examen initial objectivait une polyarthrite périphérique non érosive, un rash malaire, une photosensibilité, des ulcérations buccales, un syndrome sec, un épanchement pleural et péricardique, une fièvre à 39°C et un amaigrissement. L'hémogramme objectivait une anémie à 9,8 g/dl, des leucocytes à 4200/mm3 et une thrombocytose à 618000/mm^3^. La CRP était à 48 mg/L, la VS à 65 mm, la fibrinémie à 4,58 g/L. Les anticorps antinucléaires, les auto-anticorps anti-DNA natifs, anti-Sm, anti-U1RNP, anti-SSA/Ro et SSB/La étaient positifs. Les radiographies des mains et des pieds étaient normales. Le diagnostic de LES était facilement posé devant la présence simultanée de 7 critères de l'ACR. Le score d'activité SLEDAI était à 21. La CRP au 5ème jour d'hospitalisation était à 96 mg/L, la VS à 80 mm et la fibrinémie à 8,01 g/L. Les explorations répétées (goutte épaisse, hémoculture, uroculture, coproculture, tubage gastrique) à la recherche d'une infection n'avaient isolé aucun germe. La CRP à J15 s'élevait à 192 mg/L et la thrombocytose s'accentuait à 771000/mm^3^, malgré une antibiothérapie à large spectre et un traitement antipalustre empirique. Devant ce tableau, un SAM a été évoqué. Les examens biologiques retrouvaient une hypertriglycéridémie à 1,58 g/l, une hyperferritinémie à 3099 mg/l, et un taux de LDH augmenté à 693 U/l. Le myélogramme montrait une moelle riche avec de nombreux macrophages et des images d'hémophagocytose, évocatrices de SAM. Des bolus de methylprednisolone, 600 mg ont été institués pendant 3 jours, suivi par la prednisone per os, 60 mg/j, associée à l'hydroxychloroquine, 400 mg/j. L'apyrexie a été constatée dès le 2ème jour du bolus (J17). Une cystite a été le seul évènement indésirable. Avec peu de facteurs de mauvais pronostic [2], l'évolution a été spectaculaire (score SLEDAI = 6, disparition du syndrome inflammatoire). Après 22 mois de suivi, sans poussée, ni évènement intercurrent, est apparu un sepsis sévère, fatal pour notre patiente avant son transfert dans notre service.

## Discussion

Ce cas clinique n'était pas suggestif d'un SAM. En effet, nous n'avions pas de bicytopénie, ni de pancytopénie. L'hémogramme de notre patiente mettait en évidence une anémie, un taux normal de leucocytes et une thrombocytose. Au cours du LES avec SAM, l'anémie et la leucopénie sont constantes [3]. La thrombopénie est présente chez 75% des patients [3]. À notre connaissance, aucun cas de thrombocytose n'a été rapporté au cours d'un SAM compliquant un LES. Le rôle de la thrombocytose dans la pathogénie du SAM chez notre patiente est inconnu. Nous évoquons l'hypothèse selon laquelle la thrombocytose pourrait être présente au début de l'hémophagocytose, consécutive à une importante réaction inflammatoire, telle que celle présente dans les poussées de LES. La normalisation du taux des plaquettes voire la thrombopénie serait alors l'étape suivante, liée à une aggravation de l'hémophagocytose et/ou l'apparition d'une splénomégalie.

Le diagnostic de SAM a été rapidement posé chez notre patiente, quelques jours après celui du LES, qui évoluait depuis 11 mois. L'âge jeune de notre patiente, le diagnostic rapide et le traitement précoce pourraient expliquer l'évolution initialement favorable de la maladie chez notre patiente. Le rôle de la thrombocytose dans cette évolution est inconnu. La description d′autres cas de thrombocytose au cours d'un SAM compliquant un LES pourra permettre de confirmer notre hypothèse physiopathologique, et de préciser le rôle d'autres discordances telles que des taux de CRP et de fibrinémie élevées dans notre observation; contrastant avec l'hypofibrinémie et un taux de CRP normal ou peu élevée, fréquemment observés au cours du LES associé au SAM [3].

## Conclusion

En conclusion, l'existence d'une thrombocytose, l'absence de bicytopénie ou de pancytopénie n'élimine pas ce diagnostic de SAM, en particulier au cours du LES.
